# Some Impurities of Vended Milks

**Published:** 1909-03

**Authors:** I. Walker Hall

**Affiliations:** Professor of Pathology at University College, Bristol; Pathologist to the Bristol Royal Infirmary


### SOME IMPURITIES OF VENDED MILKS.

BY

I. WALKER HALL, M.D.,

*Professor of Pathology at University College, Bristol;*
*Pathologist to the Bristol Royal Infirmary.*

THROUGH the courtesy of a colleague, it has been possible to examine some 240 samples of Bristol milk collected under good conditions. Advantage was taken of this opportunity to determine the cell contents, the amount of dirt, and the treatment of the milk prior to exposure for sale.

1. *The cell contents of vended milks.*

A considerable amount of evidence has been accumulated upon the leucocytes of milk obtained direct from healthy and diseased cows. It shows that milk from the same cow varies from day to day, and that the hours of milking, pregnancy and drying off, all influence the cell output. When mastitis is present the number of leucocytes is materially increased, and probably a large percentage are pus cells. It has been found impossible, however, to distinguish a breaking-down leucocyte from a pus cell. Attempts have therefore been made to establish an arbitrary standard as to the number of leucocytes sufficient to warrant a diagnosis of pus. Few of these standards have been accepted, but Savage, who has worked long at this question, considers that a count of more than 800 leucocytes per c.cm. calls for an inquiry into the source of the milk.

The value of these counts is not definitely settled, but there are indications that the method may attain general application. When this is the case, an augmented Public Health staff will direct attention first to vended milks, and later to the milks of individual cows. It seemed desirable, therefore, to investigate the condition of vended milks from this standpoint, and to determine how far the obvious complicating factors might influence the results.

The method employed was that advocated by Savage (*J. Hygiene*, 1906, vi, 123), and the average time elapsing between the milking and examination was about twelve hours.

For purposes of comparison, and in order to obtain some idea of the action of time on the breaking up of the leucocytes, several samples of human milk were counted. The figures quoted in Table I. state the counts of the cell contents of human milk, and the percentage decrease of the cells when the milk is kept in sterile vessels. The increased number of cells in the milk obtained from a case of mammary abscess is also indicated.

TABLE I.

CELL CONTENTS OF HUMAN MILK.

| Day after parturition. | | Time after withdrawal, 5 hours. | | Time after withdrawal, 15 hours. | | Time after withdrawal, 45 hours. |
|---|---|---|---|---|---|---|
| | | per c.cm. | | per c.cm. | | per c.cm. |
| 4 | .. | 75 | .. | 50 | .. | 45 |
| 5 | .. | 50 | .. | 40 | .. | 30 |
| 7 | .. | 70 | .. | 60 | .. | 50 |
| 11 Case of mammary abscess | .. | 170 | .. | — | .. | 120 |

Counts made from cows at a farm in the neighbourhood yielded similar results.

When milk is exposed for sale, the debris and cells tend to be deposited at the bottom of the receptacle, while the cream rises to the top. Table II. shows this difference, and indicates that for the enumeration of cells it is necessary to stir thoroughly before taking the sample of milk.

TABLE II.

CELL CONTENTS OF VENDED MILKS.

| | Number of leucocytes per c.cm. |
|---|---|
| Upper layers .. .. | 31.8 |
| | 119.3 |
| | 87.5 |
| | 43.7 |
| Lower layers .. .. | 1718 |
| | 839 |
| | 2883 |
| | 8034 |
| | 3560 |

If we accept the arbitrary standard of 800 leucocytes per c.cm. laid down by Savage for freshly-drawn milk, and allow for the average breaking down of cells, we arrive at something like 500 as a working standard for vended milk. In such a case, fourteen of the milks (6.5 per cent.) whose counts are recorded in Table III. deserved further inquiry with regard to the cause for the increase.

TABLE III.

CELL CONTENTS OF VENDED MILKS.

| Number of leucocytes per c.cm. | Number of milks examined. |
|---|---|
| Under 20 | 137 |
| 20–40 | 18 |
| 40–60 | 10 |
| 60–100 | 13 |
| 100–500 | 22 |
| 500–1000 | 5 |
| 1000–2000 | 3 |
| Over 2000 | 6 |

With these mixed milks it has to be remembered that a high cell content in the milk from one cow may be modified by by a very low one from another animal, so that as a general rule an average will result. When, therefore, the count exceeds the average the indications for inquiry are quite definite. On the other hand, this averaging permits the overlooking of a number of milks with high counts, and the percentage of questionable milks thus appraised is far too low.

There is much to be learned from the microscopical characters of the cells and organisms of milk; but it is, after all, a sorry substitute for the continuous inspection of the animals and their attendants which our laws ought to enforce. The proverbial straining at gnats and swallowing of camels is well exemplified in this matter, for we possess a magnificent organisation for detecting trifling additions of water and almost harmless preservatives, but concern ourselves little with the pus, infective organisms, objectionable fæcal dirt, hairs, animalcules, and sputum which have become the usual constituents of the milk supplied to large cities. It is at the source of the milk that our energies should be expended, and every sample collected for compliance with the Adulteration of Foods Act is a glaring impeachment of our national neglect to control our milk supply by an adequate number of sufficiently-empowered trained inspectors, and of our cowardliness in postponing legislation on a subject of supreme importance to the health of the people.

2. *Dirt in milk.*

The standards for the determination of the amount of dirt in milk vary considerably. In this instance the method followed has been that suggested by Délépine (*Med. Chron.*, 1908, xlvii, 473). It is a remarkable tribute to the recent advances in milk inspection that in Manchester and several large cities the percentage of dirty milks has fallen from 12.5 in 1896—1900 to 4.9 in 1906.

TABLE IV.

THE INCIDENCE OF AN EXCESSIVE AMOUNT OF DIRT IN MILK.

| Date, 1908. | Number of milks examined. | Number of milks containing excessive amount of dirt. | Per cent. |
|---|---|---|---|
| January .... | 29 .... | 5 .... | 7.2 |
| April .... | 7 .... | 2 .... | 28.5 |
| May .... | 14 .... | 2 .... | 14.2 |
| June .... | 44 .... | 11 .... | 25 |

3. *Acidity of milk.*

The following table shows the amount of acidity developed in summer milk. The average acid found in locally pasteurised milk is about 12 D/N alkali per 50 c.c. The increased acidities here recorded afford an indication of the bacterial growth in the milk exposed for sale.

TABLE V.

THE ACIDITY DEVELOPED IN MILK PURCHASED DURING MAY, JUNE AND JULY.

Average normal acidity in terms of D/N alkali—12 D/N per 50 c.c. milk.

| | | | | | | | | |
|---|---|---|---|---|---|---|---|---|
| 42 | samples | yielded | an acidity of | 25 | D/N | alkali | per | 50 c.c. milk. |
| 18 | ,, | ,, | ,, | 30 | ,, | ,, | ,, | ,, |
| 7 | ,, | ,, | ,, | 49 | ,, | ,, | ,, | ,, |
| 6 | ,, | ,, | ,, | 50 | ,, | ,, | ,, | ,, |

4. *Treatment of milk before exposure for sale.*

Following the methods recommended by Proskauer, Seligmann and Croner, in their admirable report upon the Danish milk sold in Berlin (*Ztschr. f. Hyg.*, Bd. 75, 1907), of seventy-three milks examined during May, June and July, 1908, at least 17.8 per cent. of the milks had been heated to over 80° C. prior to sale. This fact suggests that a considerable amount of badly cooled or old or doubtful milk was then on the market.

TABLE VI.

THE TREATING OF MILK PRIOR TO SALE (73 Samples examined).

| Heated over 80° C. | | Residual Coagulation test. | | Oxydase test. |
|---|---|---|---|---|
| 13 | .. | 60 | .. | negative in 29 samples. |

It may be safely inferred that even the most sceptical will admit that such results as these merit the attention of the health authorities. The tendency of modern hygiene is to deal with the sources of evils as well as with the evils themselves, and the question of milk supply, rather than milk adulteration, demands our present serious attention.

PASTEURISATION AND OTHER METHODS OF PURIFYING MILK.

The advantages and disadvantages of Pasteurisation may be summarised as follows :—

Pasteurisation at 60° C. for twenty minutes kills non-sporing pathogenic microbes.

Pasteurisation at 60° C. for twenty minutes does not destroy all the ferments.

Milk Pasteurised at 60° C. for twenty minutes is a good food for children over 3 years of age.

Pasteurised milk contains a large number of spore-bearing and often dangerous microbes.

Pasteurised milk is a better medium for bacterial growth than is raw milk.

Pasteurised milk is not a good food for children under 3 years of age.

Pasteurised or purified milk is not so good as pure milk.

Pasteurisation does not prevent contamination ; the heated milk must be used more rapidly than raw milk.

Pasteurisation perpetuates ignorant and uncleanly collection and transport of milk.

Theoretically, Pasteurisation cannot be recommended. Practically, it is the best safeguard against unclean milk which we possess ; but it is a safeguard only because we do not control the sources of our milk. In this way it has constituted a serious barrier to progress, for the name has become a fetish. Why in these days is it necessary to reiterate that the process destroys the natural bactericidal characters of milk, does not kill off all the bacteria, nor remove all the filth of the farmyard ? Why should the producer be asked to keep his cows and attendants clean and his herd free from tuberculous cattle ? Because we are so frequently told that " everything that gets into the milk is removed, neutralised or killed during Pasteurisation." This is the attitude which is so widely adopted, but it is a wrong one. Pasteurisation is a good method for dealing with dirty milk, but it is not infallible. Its efficacy is only proportional to the cleanliness of collection.

It may be of value to here refer to the valuable work recently carried out at the instance of the joint Committee of the Councils of the County Boroughs of Bradford, Hull, Leeds, Rotherham, Sheffield, and the East and West Ridings of Yorkshire by Dr. Orr. He has shown that it is at the farm where the larger number of organisms and amount of dirt is added to the milk, his examinations made upon the same samples of milk at the different stages of their transfer from the animal to the consumer proving that during railway or car transit, and at the retailer's premises, only small amounts of organisms are added.

Bearing these findings in mind, I have endeavoured to define for myself the attitude of Bristol purchasers of milk, and, thanks to the kindnesses of several colleagues, I am able to append the conditions of the purchase of milk at some of our large institutions. These details have been arranged in Table VII upon the lines of the plan devised by the Liverpool Medical Institution, and for the opportunity to avail myself of this document I am indebted to Drs. Shingleton Smith and Emrys-Roberts.

TABLE VII.

CONDITIONS OF PURCHASE OF MILK BY SEVERAL BRISTOL INSTITUTIONS.

| | A | B | C | D | E | F |
|---|---|---|---|---|---|---|
| Source .. .. .. .. | Dairy | Farm | Dairy | Farm | Dairy | Dairy |
| Quality of milk .. | Pasteurised | Fresh milk | Pasteurised | Fresh milk | Pasteurised "special" | Pasteurised "special" |
| Cooling and transit | No mention | No mention | No mention | No mention | Partial | Partial |
| Buildings, sanitation, water supply | No mention | No mention | No mention | No mention | Inspected | Inspected |
| Attendants' regulations | No mention | No mention | No mention | No mention | Partial | Partial |
| Tuberculosis and tuberculin test | No mention | No mention | No mention | No mention | No Inspection | No Inspection |
| Treatment of milk at Hospital .. | Unused portion boiled | Pasteurised | None | None | Inspected on arrival | Inspected. |
| Methods of enforcing contract | — | — | — | — | None — | None — |

The point that has been advanced in connection with Pasteurisation is well borne out by this table. The layman is acting up to his best business knowledge when he contracts for Pasteurised milk. If he were made aware that the insertion of other clauses in his contract would result in a purer milk supply, there is not a shadow of doubt but that he would at once adopt them. Were it also made clear to him that a cleanly collected, rapidly cooled milk, delivered in cooled cans, is a much superior milk to the ordinary Pasteurised commodity, he would certainly use the extent of his purchasing powers to obtain the conditions deemed necessary. Table VIII (taken from the work of Evans and Cope, *Univ. Penna. Med. Bull.*, 1908, xxi, 264 (clearly demonstrates that unheated milk restrains the growth of added organisms, while heated milks form a more favourable medium for bacterial proliferation.

TABLE VIII.

THE PERCENTAGE INCREASE OR DECREASE OF BACTERIA WHEN ADDED TO STERILE COLLECTED MILK UNDER VARYING CONDITIONS.

STERILE MILK.

| The addition of | Unheated. | Heated to 55° C. | Frozen. | Pasteurised 68° C. for 20 minutes. | Sterilised for 30 mins. at 100° C. |
|---|---|---|---|---|---|
| | per cent. | per cent. | per cent. in 4 HOURS. | per cent. | per cent. |
| Streptococci .. .. | −3.5 | −20 | −7 | +25 | +10 |
| Staphylococci .. | −20 | +4 | +40 | +15 | +100 |
| B. Coli Comm. .. | −40 | +10 | — | +100 | +2000 |
| B. acidi lactici .. | +6 | +250 | +55 | +1000 | +3500 |
| B. subtilis .. .. | +5.5 | +2.5 | +20 | +85 | +450 |

The mere diffusion of this information in the Press will not evoke much interest. A better result might be expected if it were possible to arrange for a meeting with the large purchasers of milk. At such a conference the many difficulties connected with milk production and supply might be fully discussed, and the ventilation of various views on the subject could not fail to broaden the general conception of the question. The results of the conference would then gain a wider consideration by the general public, would assist the local Health Committee in their prospective arrangements for scientific aid in hygienic problems, would guide the Medical Officer of the Local Government Board in his ideas for the framing of the much wanted Act for the regulation of milk supplies, and would constitute an important advance towards the education of those who transfer the milk from the cow to the consumer.

I am glad to have the opportunity to express my thanks to Drs. E. Russell and Fleck for material, and Dr. Carey Coombs and Mr. K. Girdlestone for laboratory assistance.

---

